# RETRACTION: Naringin Stimulates Osteogenic Differentiation of Rat Bone Marrow Stromal Cells via Activation of the Notch Signaling Pathway

**DOI:** 10.1155/sci/9852061

**Published:** 2026-06-26

**Authors:** Stem Cells International

RETRACTION: G‐Y. Yu, G‐Z. Zheng, B. Chang, et al., “Naringin Stimulates Osteogenic Differentiation of Rat Bone Marrow Stromal Cells via Activation of the Notch Signaling Pathway,” *Stem Cells International*, 2016, no. 1 (2016): 7130653 (8 pages), https://doi.org/10.1155/2016/7130653.

The above article, published online on 16 March 2016 in Wiley Online Library (wileyonlinelibrary.com), has been retracted by agreement the Journal Associate Editor; Tao‐Sheng Li; and John Wiley & Sons Ltd. The retraction has been agreed due to concerns reported on PubPeer [1] with Figure 2e. Specifically, after horizontal compression, the ß‐actin bands in Figure 2e appear highly similar to the EC109/ß‐actin bands in Figure 5a of an article by different authors [2].

The authors did not respond when queried by the publisher. After assessment by the Editorial Board, the results and conclusions can no longer be considered reliable, and the article is therefore retracted.

The authors have been notified of the retraction.
